# *Fusobacterium* in the Gut–Breast Axis: Interpreting Systemic Dysbiosis in a Romanian Breast Cancer Cohort

**DOI:** 10.3390/medicina62071266

**Published:** 2026-06-30

**Authors:** Rukie Ana Maria Ahmet, Andrei Gabriel Nascu, Georgiana Cristiana Camen, Cosmin Vasile Obleaga, Cecil Sorin Mirea

**Affiliations:** 1 Doctoral School, University of Medicine and Pharmacy of Craiova, 200349 Craiova, Romania; 2 Department of Informatics, Faculty of Science, University of Craiova, 200585 Craiova, Romania; andrei.nascu@edu.ucv.ro; 3 Department of Radiology and Medical Imaging, Faculty of Medicine, University of Medicine and Pharmacy of Craiova, 200349 Craiova, Romania; 4 Department of Surgical Semiology, Faculty of Medicine, University of Medicine and Pharmacy of Craiova, 200349 Craiova, Romania; cosmin.obleaga@umfcv.ro (C.V.O.);

**Keywords:** *Fusobacterium nucleatum*, microbiome, breast cancer

## Abstract

*Background and Objectives*: *Fusobacterium nucleatum*, an oral anaerobe well-established as an onco-pathogen in colorectal cancer, is increasingly implicated in extra-colonic malignancies, including breast cancer. Despite growing mechanistic evidence, the taxonomic composition of fecal *Fusobacterium* in breast cancer remains poorly resolved, particularly regarding genus-level signals that may precede *Fusobacterium nucleatum* enrichment and their relationships with clinicopathological and demographic variables. This study aimed to quantify *Fusobacterium* (*Fs*) fecal abundance in a Romanian breast cancer cohort, assess its independence from environmental and demographic factors, and evaluate its association with disease-specific parameters, including TNM staging and BRCA mutational carrier status. *Materials and Methods*: This retrospective case–control study enrolled 99 women at the University of Medicine and Pharmacy of Craiova between October 2020 and June 2025, comprising 57 breast cancer patients and 42 healthy controls. Fecal samples underwent 16S rRNA gene sequencing targeting the V3–V4 hypervariable regions. Bacterial abundance was encoded ordinally and analyzed using the Mann–Whitney U test, the chi-squared test, Spearman’s Rank Correlation, and Kendall’s Tau test, with stratification by residential environment, age, commensal co-occurrence, and cancer-specific variables. *Results*: *Fs* abundance was highly significantly elevated in breast cancer patients relative to controls (Mann–Whitney U: *p* = 2.433 × 10^−12^; chi-squared: *p* = 1.548 × 10^−13^), with no significant associations identified with residential environment or patient age across all stratified groups. No significant correlations were identified with tumor size, lymph node involvement, or metastatic status. A statistically significant differential abundance was observed between BRCA mutation carriers and non-carriers (Mann–Whitney U: *p* = 0.00029; chi-squared: *p* = 0.00076). *Conclusions*: Fecal *Fs* abundance demonstrates cancer-specific enrichment independent of demographic and environmental determinants and is significantly associated with BRCA mutational status, positioning it as a candidate non-invasive biomarker and mechanistic contributor within the gut–breast dysbiosis axis, warranting prospective multi-center validation.

## 1. Introduction

Breast cancer remains the most commonly diagnosed malignancy in women worldwide and is a leading cause of cancer-related mortality, despite major advances in screening and systemic therapies. Traditional etiologic models emphasize germline susceptibility (e.g., BRCA1/2), endocrine exposures, lifestyle, and environmental factors. However, converging evidence from oral, gut, and breast tissue microbiome studies now suggests that microbial dysbiosis may contribute to breast tumor initiation, progression, and treatment response in a subset of patients. Within this emerging field, the oral anaerobe *Fusobacterium nucleatum* (*F. nucleatum*) has attracted particular attention as a potential onco-pathogen.

*F. nucleatum* is best known for its role in periodontal disease and its strong association with colorectal cancer (CRC), where it is consistently enriched in tumors, linked to worse prognosis, and mechanistically shown to promote tumorigenesis, immune evasion, and chemoresistance [1,2]. In CRC models, *F. nucleatum*—the principal oncogenic species among gut *Fusobacterium*—employs two principal adhesins to engage host tissue: FadA binds E-cadherin on epithelial cells, activating Wnt/β-catenin signaling to promote tumor cell proliferation and invasiveness [2], while Fap2 functions as a lectin that recognizes Gal-GalNAc glycans overexpressed on neoplastic cell surfaces, mediating selective colonization of tumor tissue [3]. In parallel, the *F. nucleatum* outer-membrane component lipopolysaccharide (LPS) engages the TLR4–MyD88–NF-κB axis, inducing the secretion of pro-tumorigenic cytokines—including IL-6, IL-8, and TNF-α—that sustain a chronic inflammatory microenvironment conducive to tumor progression [3]. Collectively, these data have led to the view of *F. nucleatum* as a prototypical microbial driver of tumor growth and immune escape in CRC and possibly other malignancies [4].

In the last decade, multiple groups have reported that the human breast harbors a distinct microbiome, with tumor tissue differing from adjacent and healthy breast tissue. Several profiling studies and systematic reviews of intratumoral microbiota now list *Fs*, including *F. nucleatum*, among taxa enriched in breast tumors compared with normal tissue, sometimes associated with more aggressive clinicopathologic features and poorer prognosis [5,6,7]. A dedicated systematic review and meta-analysis further found that oral *F. nucleatum* and periodontal disease are associated with increased breast cancer risk, with a pooled relative risk of ~1.8 for women with higher oral *F. nucleatum* levels and clinical gingivitis/periodontitis [6]. These epidemiologic data, while observational and heterogeneous, support the notion that *F. nucleatum* may act as a cross-compartment contributor to breast carcinogenesis, linking oral health, systemic dysbiosis, and mammary tumor biology.

Mechanistic work specifically focused on *F. nucleatum* and breast cancer has begun to clarify how this organism may influence the breast tumor microenvironment. A landmark experimental study demonstrated that as human breast cancer progresses, tumoral expression of Gal-GalNAc increases, facilitating Fap2-dependent binding and colonization by *F. nucleatum* in both human samples and mouse models [5]. Hematogenously administered Fap2-expressing *F. nucleatum* localized selectively to mammary tumors in mice, where it reduced tumor-infiltrating T cells and accelerated tumor growth and metastatic progression; antibiotic clearance of *F. nucleatum* reversed these effects [5]. Complementary reviews and experimental studies indicate that once within breast tissue, *F. nucleatum* can activate TLR4-driven inflammatory cascades, impair autophagy, and promote immune escape, thereby enhancing tumor cell survival and resistance to therapy [7,8,9]. A recent review in *Molecular Oral Microbiology* synthesized emerging data on routes of translocation (mammary–intestinal axis, direct nipple contact, hematogenous spread), colonization mechanisms via Fap2 and FadA, and downstream induction of matrix metalloproteinases, inflammatory mediators, and immune checkpoint engagement in the mammary microenvironment [8].

Most recently, new in vivo work has shown that *F. nucleatum* can directly promote breast cancer initiation and metastatic spread by inducing DNA damage and altering DNA repair dynamics. In mouse models and human cell systems, intraductal or intravenous exposure to *F. nucleatum* led to metaplastic and hyperplastic breast lesions, increased tumor growth, and enhanced lung metastasis, accompanied by DNA double-strand breaks and error-prone non-homologous end-joining [10]. Notably, BRCA1-deficient breast epithelial and cancer cells appeared particularly susceptible. They expressed higher levels of Gal-GalNAc, internalized more *F. nucleatum*, and exhibited greater DNA damage and tumor-promoting phenotypes, suggesting that *F. nucleatum* may synergize with inherited DNA repair defects to drive tumorigenesis [5,10]. These preclinical findings link a concrete oral pathobiont to both breast cancer risk and progression, particularly in genetically predisposed individuals.

In parallel, a growing body of microbiome work in breast cancer patients has focused on systemic dysbiosis, especially within the gut. The single-center Romanian study typifies this line of inquiry [11]. In that study, the authors compared fecal microbiota profiles from breast cancer patients and healthy controls and examined microbial signatures in relation to BRCA mutation status. They interpreted their findings within a gut–liver–estrogen axis framework, positing that intestinal dysbiosis can promote chronic inflammation, impair immune regulation, and alter enterohepatic circulation of estrogens, thereby contributing to the pathogenesis and progression of hormone-sensitive breast cancers. Although the study did not focus on specific species, it explicitly proposed systemic dysbiosis as a modifiable risk factor and highlighted microbiota profiling as a potential biomarker for risk stratification and treatment response.

Taken together, these strands of evidence suggest that *Fs* taxa should be viewed as a candidate onco-pathogen within a broader dysbiosis context, rather than in isolation, especially *F. nucleatum*. On one hand, studies emphasize broad shifts in gut community structure and function—including estrobolome-mediated estrogen recirculation and generalized inflammatory tone—as contributors to breast cancer risk [11]. On the other hand, mechanistic and breast-focused *F. nucleatum* studies reveal species-level effects: selective translocation and colonization of breast tumors, activation of TLR4 and β-catenin signaling, immunosuppressive remodeling of the tumor microenvironment, DNA damage, and potential cooperation with BRCA-related genomic instability [8]. Despite these advances, important gaps remain. Most gut–microbiome studies in breast cancer—including Ahmet et al.—have not systematically quantified *Fs* in stool or integrated oral, gut, and breast tissue sampling in the same individuals. Likewise, while emerging animal and ex vivo models provide compelling evidence that *Fs* taxa, notably *F. nucleatum*, can drive mammary tumor growth and metastasis, the prevalence, load, and functional impact of *Fs* taxa in human breast tumors across molecular subtypes and germline backgrounds are still incompletely defined [9].

In this context, there is a need to integrate *Fs* taxa into the systemic dysbiosis framework exemplified by the Romanian cohort. The present study aimed to: (i) quantify fecal *Fs* taxa abundance in a Romanian breast cancer cohort using 16S rRNA gene sequencing; (ii) assess whether *Fs* abundance is independently associated with the presence of breast cancer relative to healthy controls; (iii) determine whether *Fs* abundance is influenced by demographic and environmental variables including age and residential setting; and (iv) evaluate the relationship between *Fs* fecal abundance and cancer-specific parameters, including TNM staging, metastatic status, and BRCA mutational carrier status. By doing so, we hope to refine the conceptual model of the gut–oral–breast axis in breast cancer and to identify testable hypotheses regarding *Fs* taxa, particularly *F. nucleatum*, as a potential biomarker and therapeutic target within the broader landscape of systemic dysbiosis.

## 2. Materials and Methods

### 2.1. Methodology and Data Collection

This retrospective case–control study was carried out at the University of Medicine and Pharmacy of Craiova over a period spanning from October 2020 to June 2025. The study enrolled 99 women in total, 57 diagnosed with breast cancer and 42 serving as healthy controls, with the latter comprising individuals with benign breast pathology as well as those with no detectable breast abnormalities. Each participant was assigned to either an urban or rural residential category in accordance with Romania’s official administrative–territorial classification system. Under this framework, urban areas encompass municipalities and cities, while rural areas correspond to communes and the villages they administratively incorporate. This structured approach ensured consistent participant classification and strengthened the analytical framework for exploring potential associations between geographic residence and gut microbiota composition. It bears noting that Romania’s administrative–territorial system delineates municipalities, cities, communes, and villages on the basis of population size, economic significance, and infrastructure development.

Eligibility was restricted to women aged 18 and older, encompassing both patients with newly diagnosed breast cancer and those with benign or undetected breast lesions, provided they had not received antibiotic treatment within the month preceding fecal sampling and had not yet commenced chemotherapy or hormonal therapy. Subjects were excluded if they had experienced acute or chronic gastrointestinal conditions in the two months prior to enrollment, had documented mental health disorders, or had already initiated oncological treatment. Ethical approval was granted by the Ethics Committee of the University of Medicine and Pharmacy of Craiova (Approval Codes 199/20 September 2023 and 86/5 May 2026), and all participants provided written informed consent prior to enrollment. The study was conducted without interference with participants’ standard medical care, and no investigational treatments were administered.

Comprehensive data collection encompassed demographic and clinical characteristics, imaging findings, and pathological results, including pre-oncological workup, oncological and surgical treatment records, and histopathological and immunohistochemical reports. Whole-stool samples were collected by participants at home into plain sterile collection tubes, stored at approximately 4 °C immediately after passage, and transported to the laboratory within 3 h. On receipt, samples were homogenized, aliquoted, and stored at −80 °C until DNA extraction. Total bacterial DNA was extracted, and the V3–V4 hypervariable regions of the bacterial 16S rRNA gene were amplified by PCR using universal primers. Amplicons were purified, indexed, pooled in equimolar concentrations, and sequenced on an Illumina MiSeq platform using MiSeq Reagent Kit v3 chemistry (Illumina, Inc., San Diego, CA, USA) with paired-end 2 × 300 bp reads. Sequencing yielded a mean read depth of approximately 46,000 paired-end reads per sample before quality filtering. Microbial DNA was extracted from each sample and subjected to 16S rRNA gene sequencing targeting the V3–V4 hypervariable regions for microbiome profiling. Taxonomic assignment based on the V3–V4 hypervariable regions of the 16S rRNA gene reliably resolved the genus *Fs* but did not permit confident discrimination among closely related species within this genus. Given the well-documented predominance and oncogenic relevance of *F. nucleatum* among gut *Fs*, the detected signal was presumed to derive principally from *F. nucleatum*; however, this species-level attribution was not confirmed by independent species-specific methods, such as qPCR targeting *F. nucleatum*-associated loci. Accordingly, to avoid overstating the taxonomic resolution of our data, we refer throughout to the genus-level *Fs* taxa rather than to *F. nucleatum*, and the reported abundance values should be interpreted as genus-level estimates putatively reflecting *F. nucleatum* rather than confirmed species-level measurements.

### 2.2. Statistical and Data Analysis

**Figure 1:**
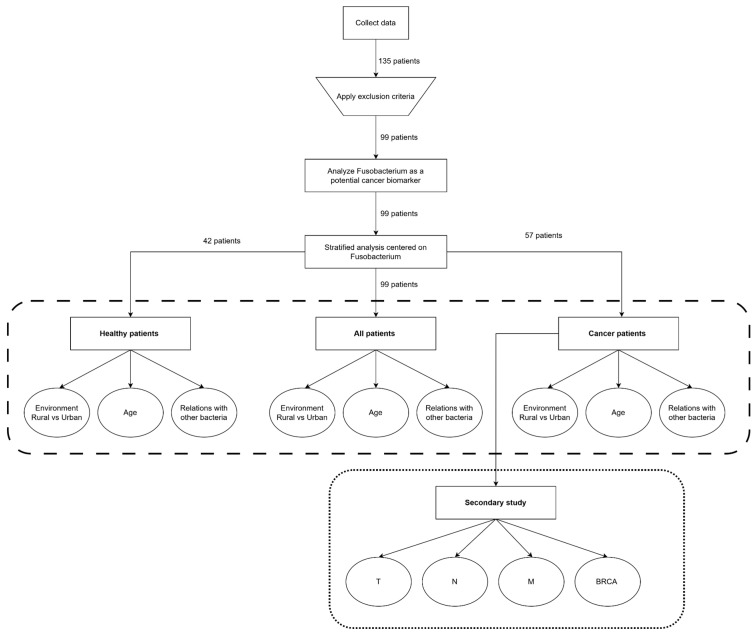
Workflow diagram of the article, comprehensive analysis of *Fs* with stratified comparison based on pathology presence as the primary study, with demographic and microbiological signature as variables. By stratifying the 99 individuals in the database, 42 healthy individuals and 57 cancer patients were obtained. Secondary study evaluates the position of *Fs* regarding cancer-specific features, such as T-tumor size, N-lymph node involvement, and M-distant metastasis (TNM staging classification).

The statistical test, graphics, and data manipulation used for this paper were performed in the Python programming language. The Python version utilized was 3.13.5, installed in an Anaconda Distribution to enable an easy way to manage the necessary modules employed in the process of investigating and exploring the relations and patterns that could occur. The Python scripts written to analyze the data used a Jupyter Notebook version 7.3.2 Integrated Developing Environment. For loading the Excel file that contained the bacterial database, pandas module version 2.2.3 was employed. Numpy module version 2.2.6 was used for fast mathematical computations, and scipy module version 1.15.3 was used for performing key statistical tests, such as Spearman’s Rank [12], Kendall’s Tau [13], the Mann–Whitney U test [14], and the chi-squared test [15]. For graph plotting, the module utilized was matplotlib version 3.10.0. The graphic in Figure 1 was designed using the online version of draw.io (https://www.diagrams.net).

Figure 1 presents the workflow of the present study. Before the statistical analyses could be run, the bacterial abundance data had to be prepared through an encoding procedure carried out in two steps. In the first step, the quantitative per-taxon abundance obtained from the 16S rRNA V3–V4 sequencing output (Section 2.1) was assigned to one of three ordinal categories—“low”, “medium”, or “high”—based on the reference range established by the laboratory for that particular bacterium in feces. Values lying within a given taxon’s normal range were labeled “medium”, while values below or above that range were labeled “low” and “high”, respectively. We chose this approach because the bacteria examined here differ widely in their baseline fecal concentrations, so the same raw value can mean very different things depending on the species. In the second step, the three ordinal categories were assigned the numerical values 1, 2, and 3, respectively, so that the non-parametric tests described in the following paragraphs could be applied. The first step in our proposed approach was to assess if there are significant differences in *Fs* abundance in relation to the pathology presence (cancer vs. healthy individuals), thus checking if a stratified analysis was further needed. This difference was assessed by primarily using the Mann–Whitney U test, and, additionally, a chi-squared test was also computed to strengthen the final results.

A stratified analysis was performed, aiming at three groups: all patients, cancer patients, and healthy individuals. For each group, three cases common to all patients, regardless of pathological status, were analyzed in relation to *Fs*’s abundance:

- For the impact of environment-related characteristics, two classes, urban and rural, were composed. To quantify the importance of this factor, two statistical tests were employed: the Mann–Whitney U test and the chi-square test.
- For quantifying the importance of discrete continuous variables, such as age, Spearman’s Rank Correlation was computed. For assuring a robustness in the evaluation, a second statistical test was performed, namely, Kendall’s Tau.
- For assessing the potential relations with other gut bacteria (“*Faecalibacterium prausnitzii*”, “*Blautia*”, “*Bifidobacterium* and *Lactobacillus*”, “*Firmicutes*”, “*Bacteroides*”, “*Clostridium species*”), encoded as ordinal categorical values, Spearman’s Rank Correlation test was performed. The initial results are further validated through the usage of Kendall’s Tau test.

A secondary study incorporating only the cancer patients was performed, taking into account pathologically related issues. The links of *Fs* abundance levels were studied in relation to TNM staging, with the following disease-specific conditions:

- For an ordinal categorical variable such as tumor (T) with stages ranging from 1 to 4 (we had no Tx, Tis, or T0 patients), a Spearman’s Rank Correlation test was computed as the first decision, followed by a Kendall’s Tau test to strengthen the results for the first decision.
- For the lymph nodes category (N), which was also considered an ordinal categorical variable, the values were 0, 1, and 2 corresponding to Nx (zero patients), N0, N1, N2, and N3 (zero patients). Here, two statistical tests were employed to evaluate potential relations: Spearman’s Rank Correlation and Kendall’s Tau.
- For analyzing the impact that metastasis presence has, a Mann–Whitney U test was utilized, followed by a chi-Squared test.
- For evaluating if the presence of BRCA mutation has a significant impact on the statistical tests, the Mann–Whitney U test and the chi-squared test were computed.

## 3. Results

**Figure 2:**
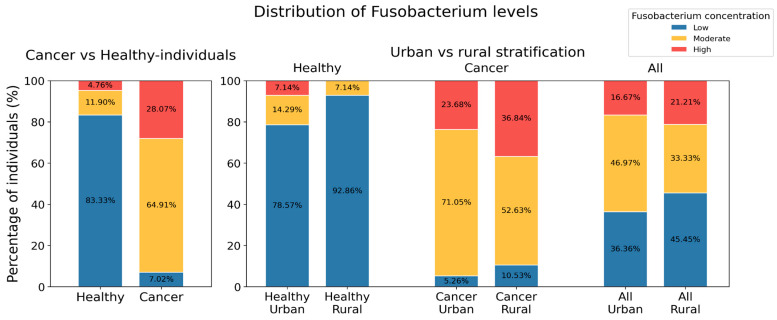
*Fs* distribution. The left panel is for cancer and healthy individuals. The right panel is the environment division (urban and rural) analyzed within the proposed stratified concept.

Analyzing *Fs* taxa, a highly significant difference in abundance level was observed between cancer cases and healthy cases by the Mann–Whitney U test, with a U statistic of 2113.5 and a significance level *p* = 2.433 × 10^−12^. A median value corresponding to a low *Fs* concentration level was observed for healthy individuals compared to a median value corresponding to a moderate *Fs* abundance for cancer patients, thus highlighting an increase in abundance driven by cancer. This finding is further supported by the chi-squared test, which gave a $x^{2}$ = 58.99 and a significance *p* = 1.548 × 10^−13^, thus indicating the dependency between *Fs* taxa and cancer. The distribution of *Fs* for cancer and healthy individuals is presented in the left panel of Figure 2. A clear distinction in abundance level can be observed, with 83.3% of healthy individuals having a low concentration of *Fs* as opposed to 64.9% of the cancer patients who had a moderate concentration. Based on this difference, a stratified analysis was justified and thus conducted with 42 healthy individuals and 57 cancer patients.

Considering the environmental factor (urban patients vs. rural patients), there are no significant differences in distribution for *Fs*, as can be observed in Table 1. Both the primary statistical test, the Mann–Whitney U test, and the secondary checking test, chi-square, did not find any meaningful association for any of the analyzed groups (healthy, cancer, all). All *p*-values in Table 1 are higher than the commonly utilized significant threshold of 0.05, thus resulting in a lack of significant associations within each group. The median value regarding the *Fs* concentration for both rural and urban patients was low for healthy individuals and moderate for both the cancer group and all patients. The distribution of *Fs* taxa’s concentration within each studied cohort, resulting from the stratification process, is presented in the right panel in Figure 2. No major shifts driven by the environment can be observed with small variations in percentages that are not identified as significant by the two statistical tests.

For a robust analysis of potential links between age and *Fs* taxa’s abundance, two statistical tests were employed: Spearman’s Rank and Kendall’s Tau. Spearman’s Rank gave no significant association, with *p*-values much above the significant threshold of 0.05 for all three groups considered in the study, as can be observed in Table 2. The correlations obtained within each group are weak, with values close to 0. The primary results computed using Spearman’s Rank are confirmed by Kendall’s Tau, with similar values for the significance factor *p* and weak correlation factor (close to 0). Both tests strongly indicated an absence of any significant monotonic relation between the two variables.

**Figure 3:**
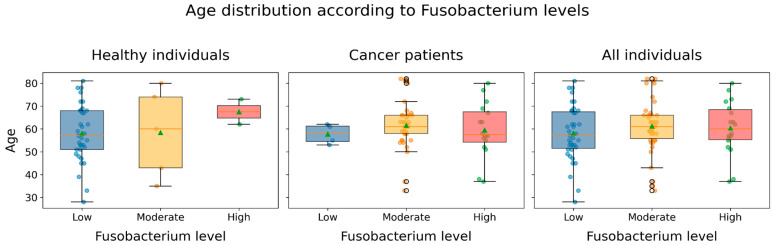
Box and whisker plots present the distribution of *Fs* for the three concentration categories, “Low”, “Moderate”, and “High”, considering the three groups resulting from stratification: healthy individuals, cancer patients, and all individuals. Each green circle represents an individual’s age, the horizontal line inside the box represents the median age within a concentration category from a group, and the green triangle represents the mean age. Each colored box represents the interquartile range (IQR), whereas the empty circles indicate an outlier value.

The distribution of age for the three levels of *Fs* concentration (low with blue, moderate with yellow, and high with red) was visually assessed by employing a box and whisker plot for each group (healthy, cancer, and all) in Figure 3. For all three groups, a high degree of overlap can be observed between the boxes. The median value was presented with a line, and the mean value was presented with a triangle for each plot. The median has a similar value for all patients and cancer patients for each concentration of *Fs*. For healthy individuals, the median is similar for low and moderate concentrations, but it has a higher value for the high bacterial concentration, which has the fewest cases. The graphic analysis supports the results obtained by the two statistical tests, with the overlap of the distributions and the similar values for the median.

**Figure 4:**
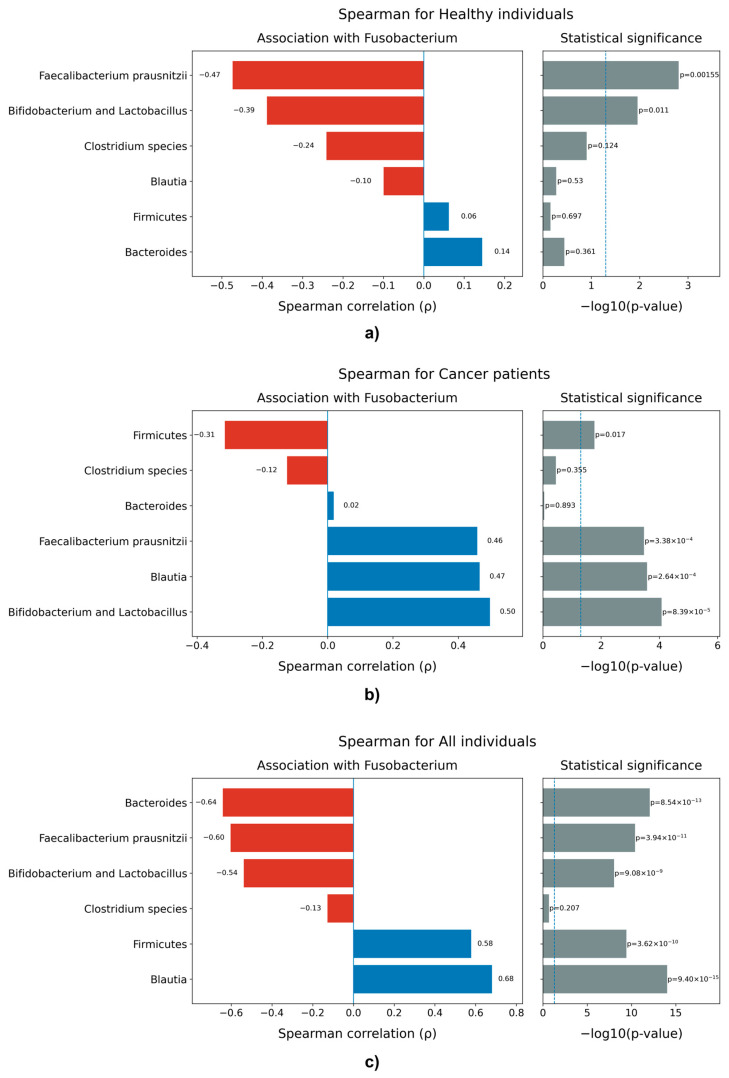
Stratified analysis for correlation and significance level between *Fusobacterium* and all other major significant gut bacterial species using Spearman’s Rank. Red represents negative correlations, and blue represents positive correlations. Any correlation is considered significant if the bar from the right panel exceeds the blue dotted line. The stratifications are made for (**a**) healthy individuals, (**b**) cancer patients, and (**c**) all patients.

For the group representing healthy individuals, most correlations of the *Fs* with other bacterial species tend to be weak or moderate in magnitude, with different levels of significance. Using the primary statistical test, Spearman’s Rank Correlation, two statistically significant associations were highlighted. *Fusobacterium* exhibits moderate inverse associations with the concentration of both *Faecalibacterium prausnitzii* (ρ = −0.473166, *p*-value = 0.001553) and *Bifidobacterium* and *Lactobacillus* (ρ = −0.388487, *p*-value = 0.011008), as can be observed in Figure 4a). These primary findings are also validated by Kendal’s Tau correlation, which indicates similar results with statistical significance for monotonic relations only for the two bacteria, as stated by the values in Table 3. No other statistically significant relations were found, with both Spearman’s Rank and Kendall’s Tau giving a *p*-value much above the significance threshold of 0.05 for all the other bacterial species within the healthy group of patients.

In the group representing cancer patients, the initial Spearman’s Rank Correlation uncovered a distinct bacterial pattern compared to the healthy cohort. Multiple and stronger associations were revealed with several correlation directions changed. One statistically significant negative correlation for *Fusobacterium* was established with *Firmicutes.* Though the *p*-value is under the threshold value of 0.05, the corresponding correlation is ρ = −0.314953, suggesting a weak inverse relation as indicated in Figure 4b. In contrast, strong, significant positive correlations between *Fusobacterium* and *Faecalibacterium prausnitzii* (ρ = 0.458254, *p*-value = 0.000338), *Blautia* (ρ = 0.465442, *p*-value = 0.000264), and *Bifidobacterium* and *Lactobacillus* (ρ = 0.497027, *p*-value = 0.000084) were identified by the Spearman’s Rank test, as can be observed in Figure 4b. No associations evaluated as significant were identified regarding *Clostridium species* and *Bacteroides*. All results obtained by Spearman’s Rank test were also confirmed by the Kendall’s Tau analysis, as suggested by the values in Table 3, thus emphasizing the existence of a set of monotonic associations with certain bacteria.

When healthy and cancer patients were combined in a single group, the statistical analysis uncovered several significant associations for *Fusobacterium nucleatum* with other bacterial species. Strong positive associations were highlighted by Spearman’s Rank, with bacteria such as *Firmicutes* (ρ = 0.578369, *p*-value = 3.624373 × 10^−10^) and *Blautia* (ρ = 0.680448, *p*-value = 9.398790 × 10^−15^), while negative significant monotonic relations were established with *Bacteroides* (ρ = −0.641390, *p*-value = 8.535222 × 10^−13^), *Faecalibacterium prausnitzii* (ρ = −0.603125, *p*-value = 3.938079 × 10^−11^), and *Bifidobacterium* and *Lactobacillus* (ρ = −0.538330, *p*-value = 9.082348 × 10^−9^), as can be seen in Figure 4c. These findings were confirmed by running the second statistical analysis, Kendall’s Tau, with the results presented in Table 3, thus emphasizing a robust and consistent set of associations. Finally, no significant associations with *Clostridium species* were identified by both statistical tests.

For the cancer patients, classified in Table 4, the relation of *Fusobacterium* with both tumor size and lymph nodes assessment was evaluated using two statistical tests: Spearman’s Rank and Kendall’s Tau. The results presented in Table 5 indicate weak negative correlations with the absence of any significant association supported by both tests. The lack of any statistical significance, with *p*-values around 0.64, much above the traditional threshold of 0.05, indicates the absence of meaningful relations between size progression and *Fs* on one side and lymph node pathological assessment and *Fs* on the other side.

The relation between metastasis and *Fs* was studied using the Mann–Whitney U test, which resulted in no significant statistical difference for the two groups (metastasis patients—M1 and non-metastasis patients—M0), with a *p*-value of 0.96825587. The initial result was strengthened by the chi-squared test, which similarly gave no statistical difference, with a *p*-value = 0.82596280, as can be seen in Table 6. The *Fs* does not present a significant difference according to metastasis criteria.

*Fs* presents a statistically significant difference when analyzing BRCA carriers versus non-BRCA carriers. These results are presented in Table 6, with *p*-value = 0.00028774 given by the Mann–Whitney U test and a *p*-value = 0.00075760 obtained by the chi-squared test. Analyzing the results of the two tests, it can be stated that the distribution of abundance levels of the *Fs* depends significantly on the BRCA mutation.

## 4. Discussion

In this study, the fecal *Fusobacterium* distribution appears significantly linked with the presence of cancer rather than with demographic or environmental factors. This enrichment, putatively attributable to *F. nucleatum*, is consistent with an active role for the organism in breast cancer pathogenesis—through its established oncogenic and immunomodulatory mechanisms—and supports its potential as a candidate biomarker for cancer presence. The results obtained by running two statistical tests, for a robust approach, suggest that *Fs* concentration level is unlikely to be significantly influenced by external (living environment) or host characteristics (age of the patients) but predominantly by cancer presence. The geographic factors do not represent a major factor in *Fs* development, as several studies also found no significant association between these taxa, notably *F. nucleatum*, and age, diet, or related epidemiologic factors in specific colorectal or fecal study settings [16,17]. *Fs* appears not to follow a gradient that is dependent on the age of the patients.

This pattern is consistent with the established biological behavior of *F. nucleatum* as a preferential colonizer of neoplastic tissue. Gal-GalNAc levels increase as human breast cancer progresses, and the occurrence of *F. nucleatum* genomic DNA in breast cancer samples correlates with high Gal-GalNAc levels, with Fap2-dependent binding demonstrated in breast cancer samples [5], which is a mechanism that inherently restricts enrichment to malignant tissue rather than distributing it along environmental or demographic gradients. The tumor selectivity of *F. nucleatum* colonization has been previously noted beyond the colorectal setting; *F. nucleatum* is enriched in breast tumor tissue compared with matched healthy tissue and has been shown to promote mammary tumor growth and metastatic progression in mouse models. From a clinical utility standpoint, oral *F. nucleatum* species are a risk factor for breast cancer development, thus elevating their biomarker potential. The fecal detection approach used in this study further expands this biomarker potential into a non-invasive sampling context. The microbiome has emerged as a significant biomarker and modulator in cancer development and treatment response, with bacterial communities within tumors displaying specificity to tumor types, a principle that the demographic independence of *Fs* in this cohort directly exemplifies. Collectively, these findings support the notion that fecal *Fs* abundance—presumed to derive principally from *F. nucleatum*—reflects the oncological state of the host rather than its epidemiological context, rendering it a promising candidate for early, context-robust cancer detection [10,18,19].

The negative correlations revealed in healthy individual cohorts of three studied bacterial species, *Faecalibacterium prausnitzii*, *Bifidobacterium, and Lactobacillus*, with *Fusobacterium*, are common and consistent based on the determined roles they have as components of the microbiome of the gut. Many of the associations in this group can be classified as having a moderate correlation with a limited number of bacterial species, presenting a significant correlation, which can indicate the existence of a balanced microbial signature in which *Fusobacterium* presents many codependencies. These results are used to create a baseline for comparison with the cancer cohort, where the disease influences the evolution, links, and developments of the existing bacteria [20,21].

The finding of positive associations in cancer cohorts between *Fs*, presumably *F. nucleatum*, with bacterial species that are traditionally viewed as beneficial, such as *Faecalibacterium prausnitzii*, *Bifidobacterium*, and *Lactobacillus*, represents an intriguing result [20,21,22,23,24]. The emergence of new bacterial dependencies may be interpreted as microbial disruption of the gut under the influence of disease progression. In this context, protective bacterial species can coexist with *Fs* under the condition of a generated dysbiosis or are unable to accomplish their protective role and their normal positive behavior. Restructuring in microbiome signature can be observed with the increase in both magnitude and the number of correlations between bacterial species and *Fs* compared to the healthy cohort. These findings may indicate a shift to a more interdependent microbial interaction as opposed to a more balanced/independent bacterial community, as was observed for healthy individuals.

In healthy subjects, *Fs* displayed inverse associations with *Faecalibacterium prausnitzii* and *Bifidobacterium/Lactobacillus*, consistent with an ecologically balanced gut environment where commensal competitors constrain pathobiont expansion. The presence of anti-inflammatory strains, such as *Faecalibacterium prausnitzii*, has been linked to protective effects against inflammatory diseases, whereas its reduced abundance correlates with higher inflammation and potential cancer risk, and breast cancer patients had significantly lower *Bifidobacterium* and *Lactobacillus* counts than controls, supporting the potential involvement of these taxa in breast cancer pathophysiology. In the cancer cohort of the present study, however, these relationships reversed to positive correlations—a striking finding that may reflect a profound restructuring of the gut ecosystem under cancer-associated dysbiotic conditions. This structural rearrangement is partially supported by evidence that an increased abundance of *Faecalibacterium prausnitzii* and *Blautia* spp. is noted in advanced-stage breast cancer patients [25], suggesting that in the dysbiotic cancer microenvironment, taxa classically considered protective may undergo co-expansion alongside pathobionts rather than serving as competitors to them.

The absence of statistically significant correlations between *Fs* abundance and tumor size, lymph node involvement, or metastatic status in the cancer subgroup of this study is a nuanced finding that merits careful contextual interpretation. Evidence from the colorectal literature suggests this dissociation is not unexpected. There were no significant differences in *Fs* taxa abundance, particularly *F. nucleatum*, across different age, sex, tumor stage, location, and tumor marker groups [26] in a CRC cohort, and a meta-analysis confirmed that high abundance of *F. nucleatum* was not associated with the overall TNM stage of CRC [27], despite being correlated with poor survival outcomes. This pattern has been interpreted as consistent with a role for *F. nucleatum* in early carcinogenic initiation rather than in the governance of advanced disease burden. Indeed, in the colorectal setting, the abundance of *F. nucleatum* is gradually increased from normal to precancerous adenomatous lesions to carcinoma, suggesting a significant role in the early tumor progression of CRC [28], which implies that its enrichment may be a feature of the cancer-permissive microenvironment rather than a direct correlate of tumor bulk or anatomical spread. Fecal *F. nucleatum* detection has developed into a new non-invasive screening strategy with demonstrated diagnostic performance, and significant correlations with lymph node metastasis have been reported in some cohorts [29], yet other studies and the present data converge on stage-independence at the level of tumor and nodal assessment. This dissociation may also reflect statistical limitations inherent to the cancer subgroup sample size, and larger longitudinal breast cancer cohorts stratified by molecular subtype will be necessary to determine whether *F. nucleatum* enrichment is indeed confined to the early carcinogenic window or whether associations with advanced disease emerge under specific conditions.

The statistically significant difference in *Fs* abundance between BRCA-positive and BRCA-negative patients in this cohort is arguably the most clinically actionable secondary finding of the study, and it is mechanistically grounded in recently published molecular evidence. *Fs*, most notably *F. nucleatum*, might promote breast cancer progression through activating the Toll-like receptor 4 pathway and by suppressing the immune system, resulting in cell growth and treatment resistance through autophagy as well as immune evasion [7]—pathways particularly relevant in BRCA-deficient tumors that already harbor compromised DNA repair and altered immune landscapes. These convergent data suggest that BRCA mutational status may not only predispose to breast cancer through genomic mechanisms but may also condition the tumor microenvironment in ways that selectively favor *F. nucleatum* colonization, transforming an oral commensal into a potent co-carcinogen in genetically susceptible hosts [30,31].

The detection of a cancer-associated *Fs* signal in fecal rather than intratumoral samples supports a conceptualization of gut dysbiosis as a systemic phenomenon that accompanies and potentially precedes mammary carcinogenesis rather than being confined to the local tumor microenvironment. The biological plausibility of this link is substantiated by the entero-mammary axis. Fecal transplants altered both the gut and mammary tumor microbiota populations in murine models, suggesting a bidirectional link between gut and breast microbiomes, with high-fat-diet-induced pro-tumorigenic effects transmissible via fecal transplant to control-diet animals [32]. This axis implies that gut microbial composition can directly influence the mammary tumor microenvironment through systemic translocation of bacteria and their metabolic products. Distinct microbial patterns have shown promise as non-invasive diagnostic and prognostic biomarkers, supporting patient stratification and risk assessment based on microbiota composition [33], and the use of fecal sampling as a cancer detection strategy is further reinforced by evidence that coordinated gut microbiome, phageome, and metabolome alterations characterize breast cancer, with a machine learning model integrating microbial and predicted metabolic features, achieving area under the curve values of 0.78 in a discovery cohort and 0.73 in an independent validation cohort [34]. Taken together, these data suggest that stool-based *F. nucleatum* quantification may function as a remote systemic readout of the oncological state of the breast, a concept that, if validated prospectively, could have implications for the development of non-invasive, low-cost breast cancer screening adjuncts.

Several methodological constraints inherent to the design and analytical framework of this study must be acknowledged in the interpretation of its findings. First and foremost, the retrospective single-center case–control design precludes the establishment of temporal relationships between *Fs* taxa’s abundance and breast cancer onset or progression; whether the observed microbial enrichment antecedes, accompanies, or is consequent to malignant transformation cannot be determined from cross-sectional fecal sampling alone, and prospective longitudinal cohorts with repeated microbiome assessments will be required to resolve this fundamental question of directionality. Second, the total sample size of 99 participants, while adequate for primary group-level comparisons, constrains the statistical power of secondary stratified analyses, particularly those involving further dichotomization of the cancer subgroup by BRCA mutational status, TNM staging, and metastatic disease; these secondary findings should accordingly be regarded as preliminary hypothesis-generating signals rather than definitive conclusions. Third, fecal sampling, while non-invasive and methodologically tractable, captures the luminal gut microenvironment and does not permit direct inference regarding intratumoral, mammary, or oral microbial compartments; the extent to which fecal *Fs* taxa, mainly *F. nucleatum*’s abundance, reflects local breast tissue colonization—the mechanistically relevant compartment—remains an open and empirically unresolved question. A methodological limitation concerns the need for *F. nucleatum* detection and quantification. In this study, *Fs* abundance was derived from 16S rRNA gene sequencing targeting the V3–V4 hypervariable regions, which provides adequate taxonomic resolution for community-level profiling but has inherent constraints when applied to species-level quantification of a single taxon. Orthogonal validation using quantitative PCR (qPCR) with species-specific primers targeting *F. nucleatum-associated* genomic loci—such as the *fap2* or *fadA* virulence genes or a *F. nucleatum*-specific 16S rRNA target—was not performed in the present study. Such validation would substantially strengthen the specificity and quantitative fidelity of the abundance data reported here.

## 5. Conclusions

This study demonstrates that *Fs* may hold a biologically and clinically significant role within the systemic dysbiotic landscape of breast cancer. Its highly significant fecal enrichment in cancer patients relative to healthy controls—confirmed by two independent statistical methods and independent of residential environment and patient age—establishes that its abundance is governed by the oncological state of the host rather than by demographic or ecological gradients, consistent with Fap2-mediated preferential colonization of Gal-GalNAc-expressing neoplastic tissue. The inversion of co-occurrence patterns between *Fs* and protective commensal taxa across the healthy and cancer cohorts further reflects a fundamental cancer-driven restructuring of gut microbial interaction networks. Its dissociation from TNM staging and metastatic status suggests a role in early carcinogenic initiation rather than advanced disease burden, while its significant differential abundance according to BRCA mutational status—the most clinically actionable finding of this study—provides cohort-level support for the hypothesis that BRCA1-associated surface molecular alterations selectively potentiate *Fs* taxa, most probably *F. nucleatum*, colonization and its downstream oncogenic sequelae.

Collectively, these findings might make *Fs* a candidate biomarker and hypothesized mechanistic contributor within the oral–gut–breast dysbiosis axis, warranting validation in prospective, multi-center, longitudinal studies integrating multi-compartment microbiome sampling, whole-metagenome sequencing, and metabolomic profiling to establish causal directionality and evaluate the therapeutic relevance of its targeted modulation.

## Figures and Tables

**Table 1:** Stratified analysis regarding distribution differences of *Fs* taxa’s abundance in relation to external factors, such as the living environment of the patients (urban, rural).

Statistical Test		Healthy Individuals	Cancer Patients	All Patients
Mann–Whitney U	U statistic	225	330.5	1140
	*p*-value	0.2405	0.5450	0.6854
Chi-squared	x^2^ statistic	1.6285	1.9434	1.6758
	*p*-value	0.4429	0.3784	0.4326
	Degrees of freedom	2	2	2

**Table 2:** Stratified correlation analysis between *Fs* taxa’s abundance and demographic factors, such as age.

Statistical Test		Healthy Individuals	Cancer Patients	All Patients
Spearman’s Rank	ρ	0.1192	−0.0123	0.1177
	*p*-value	0.4518	0.9273	0.2455
Kendall’s Tau	τ	0.0925	−0.0081	0.0915
	*p*-value	0.4694	0.9408	0.2516

**Table 3:** Stratified correlation analysis between *Fusobacterium* and all other major significant gut bacterial species.

Statistical Test	Bacteria	Results For	Healthy Individuals	Cancer Patients	All Patients
Spearman’s Rank	*Faecalibacterium prausnitzii*	ρ	−0.473166	0.458254	−0.603125
		*p*-value	0.001553	0.000338	3.938079 × 10^−11^
	*Blautia*	ρ	−0.099662	0.465442	0.680448
		*p*-value	0.530033	0.000264	9.398790 × 10^−15^
	*Bifidobacterium and Lactobacillus*	ρ	−0.388487	0.497027	−0.538330
		*p*-value	0.011008	0.000084	9.082348 × 10^−9^
	*Firmicutes*	ρ	0.061973	−0.314953	0.578369
		*p*-value	0.696620	0.017022	3.624373 × 10^−10^
	*Bacteroides*	ρ	0.144604	0.018292	−0.641390
		*p*-value	0.360887	0.892573	8.535222 × 10^−13^
	*Clostridium species*	ρ	−0.241144	−0.124677	−0.127929
		*p*-value	0.123942	0.355461	2.069817 × 10^−1^
Kendall’s Tau	*Faecalibacterium prausnitzii*	τ	−0.454902	0.446258	−0.522043
		*p*-value	0.002603	0.000605	1.348637 × 10^−8^
	*Blautia*	τ	−0.098020	0.443086	0.643994
		*p*-value	0.523379	0.000440	1.776728 × 10^−12^
	*Bifidobacterium and Lactobacillus*	τ	−0.378074	0.476727	−0.467770
		*p*-value	0.012968	0.000204	4.756346 × 10^−7^
	*Firmicutes*	τ	0.060952	−0.303549	0.507510
		*p*-value	0.691498	0.018708	2.979521 × 10^−8^
	*Bacteroides*	τ	0.142222	0.017813	−0.587765
		*p*-value	0.354488	0.891125	1.862302 × 10^−10^
	*Clostridium species*	τ	−0.234769	−0.121413	−0.121514
		*p*-value	0.122445	0.350822	2.035945 × 10^−1^

**Table 4:** Profiles of the two subgroups of cancer cases (legend: NOS = not otherwise specified; F-C Ch = fibro-cystic change; M = malignancies; NCO = not carried out; NN = non-neoplastic lesions; DCIS = ductal carcinoma in situ; IDC = invasive ductal carcinoma; ILC = invasive lobular carcinoma; L-A = luminal A type; L-B = luminal B type; T-N = triple-negative).

Analyzed Characteristic		Number of Cancer Cases	
		Without BRCA	With BRCA
Age	≤40 years	1	3
	>40 years	43	10
Histogenetic type	NOS	21	2
	F-C Ch	0	0
	M	23	11
Histopathological diagnosis	NCO	0	0
	NN	0	0
	DCIS	13	0
	IDC	16	11
	ILC	15	2
Molecular classification	NCO	0	1
	L-A	10	3
	L-B	18	2
	HER2+	11	0
	T-N	5	7
Other pathology	Presence	8	6
	Absence	36	7

**Table 5:** Correlation between *Fs* abundance level and T and N.

Statistical Test		T (TNM Stadialization)	N (TNM Stadialization)
Spearman’s Rank	ρ	−0.11905422	0.06248142
	*p*-value	0.37773937	0.64427844
Kendall’s Tau	τ	−0.10993873	0.05817189
	*p*-value	0.38032371	0.63908865

**Table 6:** Analyzing statistical distribution differences by dichotomizing the cancer group using M and BRCA criteria.

Statistical Test		M (TNM Stadialization)	Presence of BRCA
Mann–Whitney U	U statistic	142.5	125.5
	*p*-value	0.96825587	0.00028774
Chi-squared	$\chi^{2}$ statistic	0.38241106	14.37069138
	*p*-value	0.82596280	0.00075760
	Degrees of freedom	2	2

## Data Availability

The original contributions presented in this study are included in the article. Further inquiries can be directed to the corresponding author.
